# In Freeze-All Strategy, Cumulative Live Birth Rate (CLBR) Is Increasing According to the Number of Blastocysts Formed in Women <40 Undergoing Intracytoplasmic Sperm Injection (ICSI)

**DOI:** 10.3389/fendo.2019.00427

**Published:** 2019-07-03

**Authors:** Evangelos Papanikolaou, Tatiana Chartomatsidou, Evangelia Timotheou, Petroula Tatsi, Eleftheria Katsoula, Christina Vlachou, Irene Asouchidou, Odysseas Zafeiratis, Robert Najdecki

**Affiliations:** ^1^Assisting Nature, Centre of Assisted Reproduction and Genetics, Thessaloniki, Greece; ^2^3rd Department of Obstetrics and Gynecology, Aristotle University of Thessaloniki, Thessaloniki, Greece; ^3^Medical Department, Aristotle University of Thessaloniki, Thessaloniki, Greece

**Keywords:** freeze-all policy, blastulation rate, blastocyst, cumulative live birth rate, cumulative pregnancy rate, IVF outcome

## Abstract

**Background:** Elective freezing of all embryos, followed by frozen-thawed ET cycles emerged to prevent risk of Ovarian Hyperstimulation Syndrome and to allow endometrium recovery after Controlled Ovarian Stimulation, leading to better IVF outcomes. Blastocyst Freeze-all policy can minimize the number of abnormal embryos and consequently failed ETs, but its efficacy in terms of cumulative rates has not been studied yet.

**Methods:** A prospective cohort observational study was carried out in Assisting Nature, Center of Assisted Reproduction and Genetics, in Thessaloniki, Greece from January 2014 until December 2017. 244 patients- normal or high responders- underwent COS with recFSH and Freeze-all policy with blastocyst culture. The included patients were 18-39 years and achieved clinical pregnancy and/or live birth or had all their vitrified blastocysts transferred in subsequent frozen-thawed cycles. Women were divided into four groups (group A: 1–2 blastocysts frozen; group B: 3–4; group C: 5–6; group D ≥7 blastocysts frozen) or seven groups (group I: 1–2 blastocysts frozen, group II: 3, group III: 4, group IV: 5, group V: 6, group VI: 7; group VII: ≥8 blastocysts frozen), according to the numerical range or to the absolute number of vitrified blastocysts, respectively.

**Results:** The main outcome of the study was the CLBR achieved by frozen-thawed ETs, according to the number of the vitrified blastocysts. Higher CLBR are expected, when at least 3 blastocysts are formed (group B: 65.2%) and at least 2 frozen-thawed ETs are performed, reaching highest rates (88%) by group D (≥7 vitrified blastocysts). Similarly, CLBR is significantly increasing with the absolute number of the vitrified blastocysts, ranging from 20%, when 1–2 blastocysts are vitrified (group I) to 82.4% when ≥8 blastocysts are available.

**Conclusions:** A higher number of vitrified blastocysts is associated with higher CLBR in women <40 years old- normal/high responders- following Freeze-all policy. Adopting Freeze-all strategy after blastocyst culture can contribute to improve delivery outcome after IVF, in terms of CLBR. The number of the total cryopreserved blastocysts produced might reflect the quality of the oocyte and can successfully predict the pregnancy outcome. The blastulation rate can be a robust criterion to segment or not an IVF cycle.

## Introduction

Although fresh embryo transfer is the golden standard in IVF and millions of couples worldwide achieved a live birth, huge progress has been recorded in various aspects of Assisted Reproduction. Especially, one main advancement is the improvement of culture conditions, allowing thus many fertilized eggs to proceed into blastocyst stage and secondly, vitrification of embryos has allowed excellent survival without any impact on their quality (1–3).

The concept of Freeze-all strategy has evolved due to two main drawbacks of multi-follicular stimulation for IVF. The first reason for the emerge of the Freeze-all strategy is safety. Ovarian Hyperstimulation Syndrome (OHSS) is considered one of the major causes of morbidity between women following Controlled Ovarian Stimulation (COS). The replacement of hCG with a GnRH agonist for oocyte maturation triggering limited the incidents of OHSS, especially among high risk patients (4, 5), but it is associated with lower implantation rates (6, 7). A GnRH antagonist protocol with a GnRH agonist trigger and Freeze-all strategy can prevent both early and late onset of OHSS, since the embryo transfer (ET) is delayed in a consecutive cycle.

The second reason is related to the endometrium environment after the COS. An increase in the number of the retrieved oocytes and consequently a higher number of available embryos is expected to contribute in a relative increase of the delivery rate (8, 9). However, performing fresh ET in a fresh cycle, after the COS, is correlated with lower live birth rates, if more than 20 eggs are collected (8, 10). Implantation is a process dependent on the embryo quality, on the endometrial receptivity and on their interaction (11). COS may enable excess number of oocytes to be retrieved and consequently fertilized, but at the same time leads to supraphysiological hormone levels during the follicular phase (12), impairing the endometrium receptivity (13–17). The segmentation of the IVF treatment in a stimulation cycle followed by Freeze-all strategy and a frozen-thawed embryo transfer in a subsequent cycle can eradicate the negative effects of COS and is associated with higher cumulative success rates (18–20).

However, it is apparent that Freeze-all policy, in order to be widely accepted and applicable, should firstly be proved more efficient than the classical policy so far, fresh first followed by thawed surplus embryos, in terms of cumulative delivery rates. Secondly, it has to be proved equally safe- if not better- in terms of health of the offsprings and with regards to the obstetrical outcome. Recent systematic reviews (21, 22) on babies conceived by frozen embryo transfers confirmed a lower risk of preterm delivery, small for gestational age and low birth weight. On the other hand, it has also been showed that frozen embryo transfer is associated with an increased risk of hypertensive disorders of pregnancy and large for gestational age (21, 22).

Regarding the offspring health it is not a matter of the current research, as we focused on the cumulative results of the stimulated ART cycles. Over the past years, much effort was put in determining the clinical outcomes of an IVF treatment regarding the number of the retrieved oocytes. Large studies supported that live birth rates are increasing with the number of retrieved oocytes, reaching a plateau after 10–15 oocytes (8, 23). On the other hand, a more recent study of Polyzos et al. (24) showed a continuously increase in the LBRs with the number of the oocytes, without any plateau. The study of Acharya et al. (25), based on data (2014–2015) from the American Society for Assisted Reproductive Technology Registry, showed that frozen embryo transfers benefited only patients who produced a large number of oocytes (≥15). However, both clinical pregnancy and live birth rates in intermediate responders were higher after fresh embryo transfer than after Freeze-all and similarly the same trend was observed in poor responders, as well.

Other studies on Freeze-all policy however, suggested that improved IVF results are associated not only with cases of high responders (12), but with normal responders (17, 26) and even with poor responders (27). Although this latter is still controversial (28–31).

The current prospective cohort study aims to identify the probability of pregnancy according to the total number of blastocysts frozen per group of patients, in cases which do not undergo fresh ET, but perform total freezing and segmentation of cycle at the blastocyst stage. Also, the study aims to provide information to patients regarding their chances to deliver a baby after a single ovarian stimulation in terms of cumulative delivery rates.

## Materials and Methods

### Patient Selection

**Figure 1:**
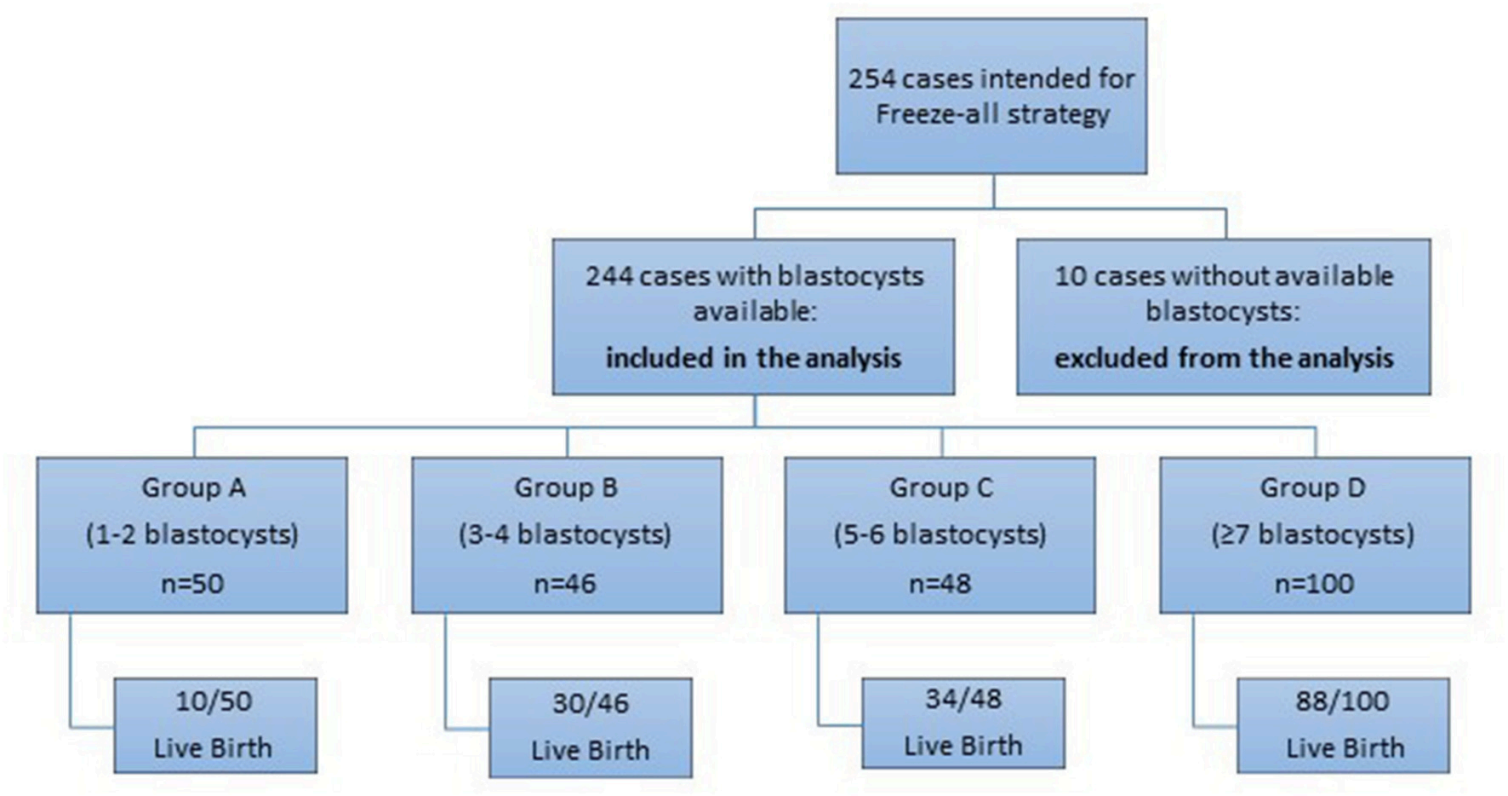
Diagrammatic depiction of the included and excluded patients of the analysis.

A prospective cohort observational study was performed in 254 women undergoing ICSI treatment from January 2014 until December 2017 in Assisting Nature, Center of Assisted Reproduction and Genetics, Thessaloniki, Greece. Among these women who intended to follow Freeze-all strategy, 10 patients had no blastocysts at the end of the embryo culture, despite that the range of the retrieved oocytes in these cases was 5–15. The rest 244 cases, which had blastocysts available for vitrification were included in the analysis (Figure 1). All the studied cases concerned couples with a female age range of 18–39 years and with fresh ejaculated sperm. The recruitment of the patients for the Freeze-all strategy was performed at the day of egg retrieval and only if the retrieved oocytes of a patient were >4. Exclusion criteria were: donor oocyte programs, PGS/PGD cycles, women with ≤ 4 oocytes per retrieval and cases with sperm retrieved by TESE. Written informed consent was signed and obtained by all the participants of the study. The study was registered in clinicaltrials.gov under the registration number NCT03463278.

### Ovarian Stimulation

All patients were treated with GnRH antagonist protocol, as part of the COS of IVF treatment. Gonadotrophins (Gonal- F®; Serono, Hellas) were administered from day- 2 of the cycle, if the hormonal levels were basal. The initial dose of the gonadotrophins was predefined at 150- 300 IU for all patients and remained fixed for 5 days. After this period, the dose could be adjusted to each patient, according to the follicular growth and the estradiol levels.

Co- treatment with GnRH antagonist started on Day 6 of the stimulation with 0.25 mg cetrorelix (Merck-Serono) or 0.25 mg ganirelix (MSD).

The final oocyte maturation was induced when at least 3 follicles of 18 mm were present during ultrasound examination. Oocyte retrieval was performed 36 h after the administration of the triggering regimen. When <14 follicles were present, rec-HCG (Ovitrelle®; Merck-Serono) was administered. Alternatively, if ≥14 follicles were present, then agonist triggering was chosen with 0.3 mg Triptorelin (Arvekap®, IPSEN).

### Embryological Data

The incubation conditions of the embryo culture were set at 6% CO_2_, 5% O_2_ and 37°C. The culture of the embryos was supported by the sequential media provided by Origio® (Cleavage medium and Blastocyst medium). Zygotes were scored on day 1 after ICSI, based on the presence of the 2 pronuclei (2PN) and their morphology (32). Embryo quality was assessed on the morning of day 3 according to the number of the cells, the equality of the blastomere size and the fragmentation rate. Embryos were also evaluated on day 5 according to the embryo scoring system of Gardner and Schoolcraft (33), regarding the formation and the morphology of the blastocysts. The quality of the blastocysts was characterized as top (grade 3-5AA, 3-5AB), good (3-5BA, 3-5BB) or fair (1-2AA, 1-2AB, 1-2BA, 1-2BB). The proportion of each embryo quality in every studied group is mentioned in Table 1.

**Table 1:** The proportion of top, good, and fair quality blastocysts vitrified in the overall blastocyst number *(N)* per each group.

**Blastocyst quality**	**Group A (1-2 Blastos frozen) *N* = 92**	**Group B (3-4 Blastos frozen) *N* = 164**	**Group C (5-6 Blastos frozen) *N* = 262**	**Group D (≥7 Blastos frozen) *N* = 952**
Top	63% (58/92)	67.1% (110/164)	80.9% (212/262)	83.4% (794/952)
Good	17.4% (16/92)	19.5% (32/164)	12.2% (32/262)	12.6% (120/952)
Fair	19.6% (18/92)	13.4% (22/164)	6.9% (18/262)	4% (38/952)

Sperm preparation and ICSI procedure were carried out as described by Van Landuyt et al. (34).

The cryopreservation of the blastocysts was conducted on the fifth or the sixth day of the culture. In the studied cases, there were no incidents of day 6 blastocysts to be included in the final vitrified blastocyst cohort. Vitrification and thawing of the cryopreserved blastocysts were performed with the Kitazato® Vitrification Kit and Kitazato® Warming Kit, respectively. The vitrification carrier used was the Cryotec straw by Cryotech® Japan.

### Endometrial Preparation for FET

None of the included cases were submitted to fresh ET. The vast majority of patients underwent estrogen replacement preparation of endometrium and only 1% underwent natural cycle synchronization for transfer. Women who followed estrogen replacement preparation of the endometrium started hormonal monitoring on day-2 of the cycle and if estradiol was <80 pg/ml, progesterone was <1.5 ng/ml and no cyst was present on the ultrasound scan then the patient could start the treatment. On this day women started 17β- estradiol (Divina®; Orion Corporation or Cyclacur®; Bayer Hellas) with 2 mg once in the night of day-2 and then 4 mg from day-3 to day-5 and increasing to 6 mg from day-6 to day-8 and then increasing again to 8 mg from day-9 to day-11 onwards. On day-11 ultrasound scan and hormone level (E2, PG) test was performed. If the endometrial thickness was >7 mm, the estrogen level >150 pg/ml and the progesterone level <1.5 ng/ml (35), then the progesterone supplementation could be started upon the planning of the embryology lab. The estrogen dose was decreased to 6 and 600 mg progesterone in 3 doses of 200 mg for 6 days were administered in order to transfer blastocysts.

When the embryo replacement was conducted in a natural cycle, the monitoring started on the first day of the cycle. An ultrasound scan was conducted on day 3–4, then on day-9 and repeated on day 11–12 along with hormone levels test. The day of the transfer was decided upon definition of ovulation day, based upon monitoring the rise of LH above 14 mIU/ml or progesterone rise above 1.5 ng/ml.

The pregnancy test was performed 14 days after the progesterone supplementation initiation. In case of a negative test, the treatment was discontinued. Alternatively, in case of pregnancy, the treatment continued until 10 weeks and then gradually the medications discontinued.

### Embryo Transfer Policy

The policy of the embryo transfer of our clinic was in accordance to the Greek law. Elective double embryo transfer was offered and allowed by law. Nevertheless, if the patient's age was <32 years old, or upon the couple's request, then single embryo transfer was chosen, although the law still was allowing double ET.

### Cumulative Rates Per Individual

With the evolution of the Freeze-all strategy, a new index for evaluating the success of an ART cycle is needed. In this study, a modified index is used, based on the proposed COMFFETI (Combined Fresh and Frozen Embryo Transfers per Individual) by Papanikolaou et al. (36), which is a binomial variable (yes or no) that reflects the achievement of a pregnancy by individual (couple), after each stimulated cycle. COMFFETI index originally includes results by both fresh (when performed) and thawed ET (when performed), obtained by a single ovarian stimulation cycle, assessing the success of both fresh and frozen cycles performed by the same woman.

The cases included in the present study did not perform fresh ET, but followed Freeze-all strategy at the blastocysts stage and subsequent frozen- thawed ETs. As a result, the cumulative results of the studied cases derive only from FET cycles. The cumulative result of “clinical pregnancy” was estimated if clinical pregnancy was achieved in any of the frozen-thawed treatments, which resulted after a single ovarian stimulation. Similarly, if any of the FET cycles of a woman resulted in a delivery of a baby, then this particular couple is considered as a case which achieved “live birth.” The total number of the studied cases was 244. All patients performed up to 3 frozen-thawed ETs, except from 6 cases which performed up to 4.

For the first part of the study, patients were divided in 4 groups, according to the number of the vitrified blastocysts at the end of the stimulation cycle. Group A consists of women with 1–2 blastocysts frozen; group B includes women with 3–4 blastocysts; group C consists of women with 5–6 blastocysts frozen and group D refers to women with 7 or more blastocysts frozen.

For reasons of further analysis, these patients were also divided in 7 groups, according to the absolute number of vitrified blastocysts after the stimulation cycle; group I: 1–2 blastocysts frozen, group II: 3 blastocysts frozen, group III: 4 blastocysts frozen, group IV: 5 blastocysts frozen, group V: 6 blastocysts frozen, group VI: 7 blastocysts frozen and group VII: more than 7 blastocysts frozen.

The success rates of the ART cycles were estimated as Cumulative Rates. Cumulative Rates are estimated as the probability of achieving a clinical pregnancy/ live birth after one single stimulation cycle by transferring the available embryos in subsequent frozen- thawed ETs.

The Cumulative Clinical Pregnancy Rate (CCPR) and Cumulative Live Birth Rate (CLBR) were estimated separately for each group of patients. The included cases either achieved a clinical pregnancy/ live birth or had all their vitrified/ thawed blastocysts transferred after completing 3 cycles of frozen-thawed ETs (except from 6 cases with up to 4 FET).

### Statistical Analysis

The data from the statistical analysis are presented as mean ± standard deviation or percentage. The statistical evaluation was performed with SPSS software (SPSS, Chicago, IL, USA). Descriptive statistics were performed for each variable and the quantitative results are performed as the mean (±SD). Means were compared by using ANOVA and t-test, while proportions between the groups were compared using chi-square test. Differences were considered significant when *p* < 0.05.

### Ethical Approval

This prospective cohort observational study was approved by the Institutional Review Board of Assisting Nature, Center of Assisted Reproduction and Genetics, Thessaloniki, Greece.

## Results

### Patients Characteristics

**Figure 2:**
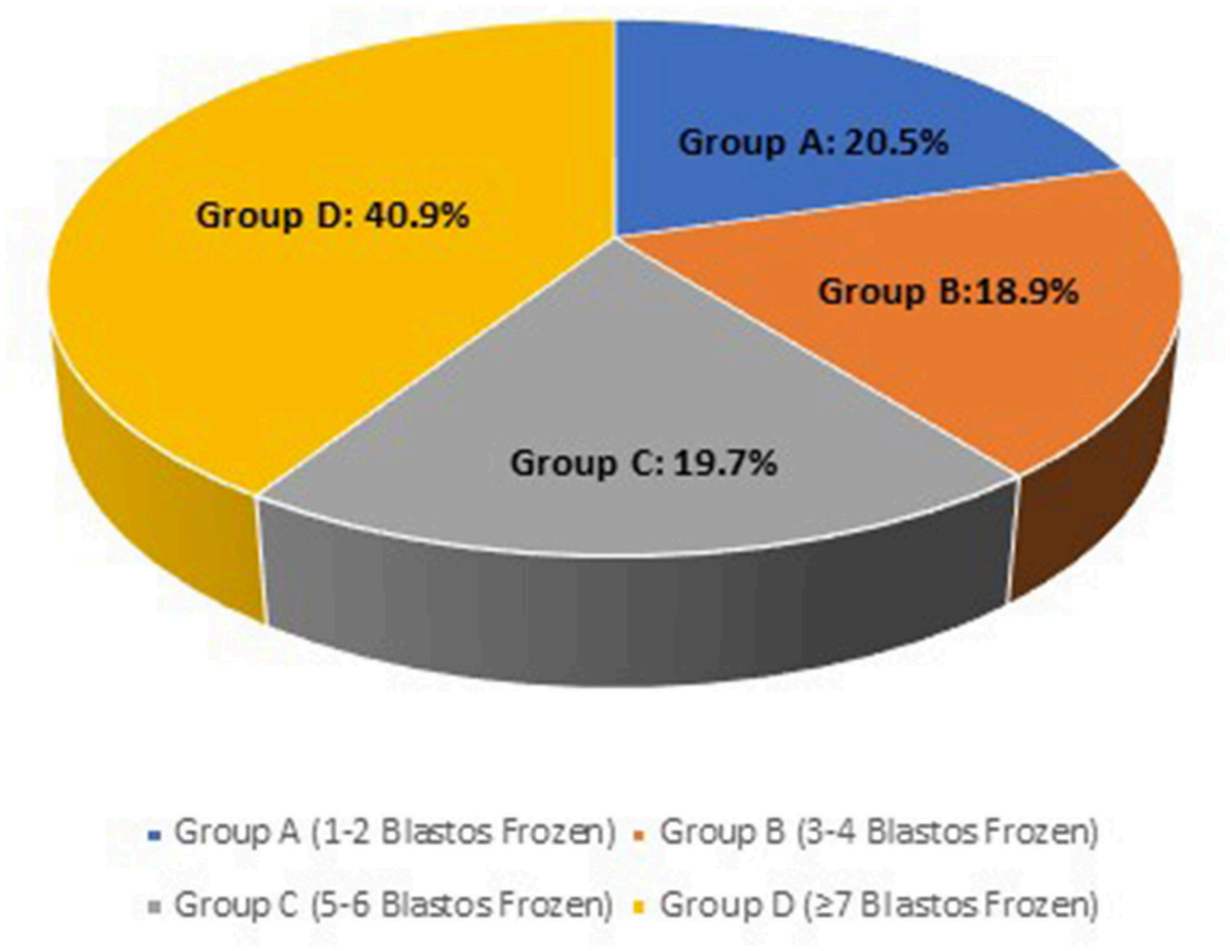
The frequency of each study group; Group A (1-2 blastocysts frozen), Group B (3-4 blastocysts frozen), Group C (5-6 blastocysts frozen) and Group D (≥7 blastocysts frozen) on the total case number.

Overall, the analysis included 244 cases divided in different groups, according to the number of the available vitrified blastocysts. Overall, the analysis included 244 cases. For the first part of the study, patients were divided in 4 groups, according to the number of the vitrified blastocysts at the end of the stimulation cycle: **Group A** (1-2 blastocysts frozen) included 50 patients (20.5%), **Group B** *(3-4 blastocysts frozen)* included 46 cases (18.9%), **Group C** *(5-6 blastocysts frozen)* comprised of 48 cases (19.7%) and the last **Group D** *(*≥*7 blastocysts frozen)* included 100 cases (40.9%) of the total population studied (Figure 2).

No complications, such as events of OHSS, were noticed after the COS of these patients. As a result, 36.1% (88/244) of the patients received HCG triggering and 63.9% (156/244) agonist triggering.

A rate of 96% of the cases underwent elective double embryo transfer, while 2% had elective single ET (elective SET) and 2% SET. In the latter case, this 2% of the women followed SET, because no more blastocysts were available.

The mean values of age, COCs (Cumulus- Oocyte Complex) and metaphase 2 (MII) oocytes are shown on Table 2. Although the mean age of the women ranged from 31.9, to 34.5, and the differences among groups did not exceed the 3 years, there was statistical difference among groups.

**Table 2:** The mean values and Standard Deviation (SD) of age, basal FSH, reason of infertility, number of COCs, number of MII oocytes, fertilization and blastulation rates, and post-thawing survival rate of the blastocyss of the groups, characterized by the statistical significance: Group A (1-2 blastocysts frozen), Group B (2-3 blastocysts frozen), Group C (5-6 blastocysts frozen) and Group D (≥7 blastocysts frozen).

		**Group A (1-2 Blastos Frozen) (*n* = 50)**	**Group B (3-4 Blastos Frozen) (*n* = 46)**	**Group C (5-6 Blastos Frozen) (*n* = 48)**	**Group D (≥7 Blastos Frozen) (*n* = 100)**	**s.s^b^ (*p*-value)**
Age^a^ mean (±SD)		31.84 (±5.21)	34.48 (±3.42)	33.96 (±3.69)	32.12 (±3.66)	P <0.05
Basal FSH mean (mIU/ml)		7.1	6.9	6.7	6.1	n.s.
Infertility reason	Male factor	40%	42%	44%	45%	n.s.
	Female factor	39%	36%	36%	32%	n.s.
	Both	21%	22%	20%	23%	n.s.
COCs mean (range)		9.80 (5–24)	11.61 (5–21)	11.96 (6–21)	18.66 (7–58)	*P* < 0.05
MII mean (±SD)		6.56 (±3.56)	8.52 (±2.84)	9.52 (±2.91)	14.66 (±5.18)	*P* < 0.05
Fertilization rate mean (±SD)		81.74% (15.66%)	79.57% (±16.04%)	85.46% (±10.35%)	86.94% (±11.39%)	n.s
Blastulation rate mean (±SD)		48.22% (±26.1%)	59.24% (±18.67%)	72.95% (±17.95)	79.55% (±20.58)	*P* < 0.05
Post-Thawing Survival rate mean (±SD)		100% (±0.1%)	100% (±0.1%)	100% (±0.1%)	100% (±0.1%)	n.s

a *Age is expressed in years*.

b *Statistical significance*.

The mean number of the COCs and the MII oocytes after the oocyte retrieval of the studied cases are also significantly different between the groups (*p* < 0.05).

As it would obviously be anticipated, the number of the COCs was significantly correlated with the number of the MII oocytes and the number of the blastocysts available for vitrification, at a significance level of 0.05.

A logistic regression analysis for the four groups was carried out with dependent variable being the achievement of live birth or not and independent variables were the age, the mean number of COCs and the blastulation rate. The analysis showed that the only variable remaining significant was the number of the COCs (*p* < 0.05).

Initially, 254 women were intended to follow Freeze-all strategy. Ten out of these women did not have any available blastocyst at the end of the embryo culture (even until the sixth day). All 10 women had 5–15 oocyte yield after the retrieval. Maturation and fertilization rate in 7 out of 10 cases were comparable with the rest of the sample. One of these 7 women got pregnant in a subsequent IVF treatment, after an additional COS cycle; 4 continued the treatment unsuccessfully so far; and the rest two accepted oocyte donation achieving pregnancy and live birth.

Maturation problem appeared in the rest 3 out of the 10 patients (16.7–53.3%). One of these women continued the trials, despite the fact that she had already undergone two IVF therapies, in other clinics, without ET. The two other cases facing maturation problem have not gotten pregnant and refused oocyte donation.

### Fertilization and Blastulation Rate

The fertilization rate was calculated by the number of the 2PN embryos divided by the total number of the MII oocytes, in each case. For the calculation of the blastulation rate, the dominator was the total number of the 2PN embryos. The mean fertilization and blastulation rates of each group are presented on Table 2. The statistical analysis showed no difference in the mean value of the fertilization rate between the groups, while there is a significant difference of the mean rates between the four groups (*p* < 0.05), regarding the blastulation rate.

### Cumulative Clinical Pregnancy Rate (CCPR) and Cumulative Live Birth Rate (CLBR)

**Figure 3:**
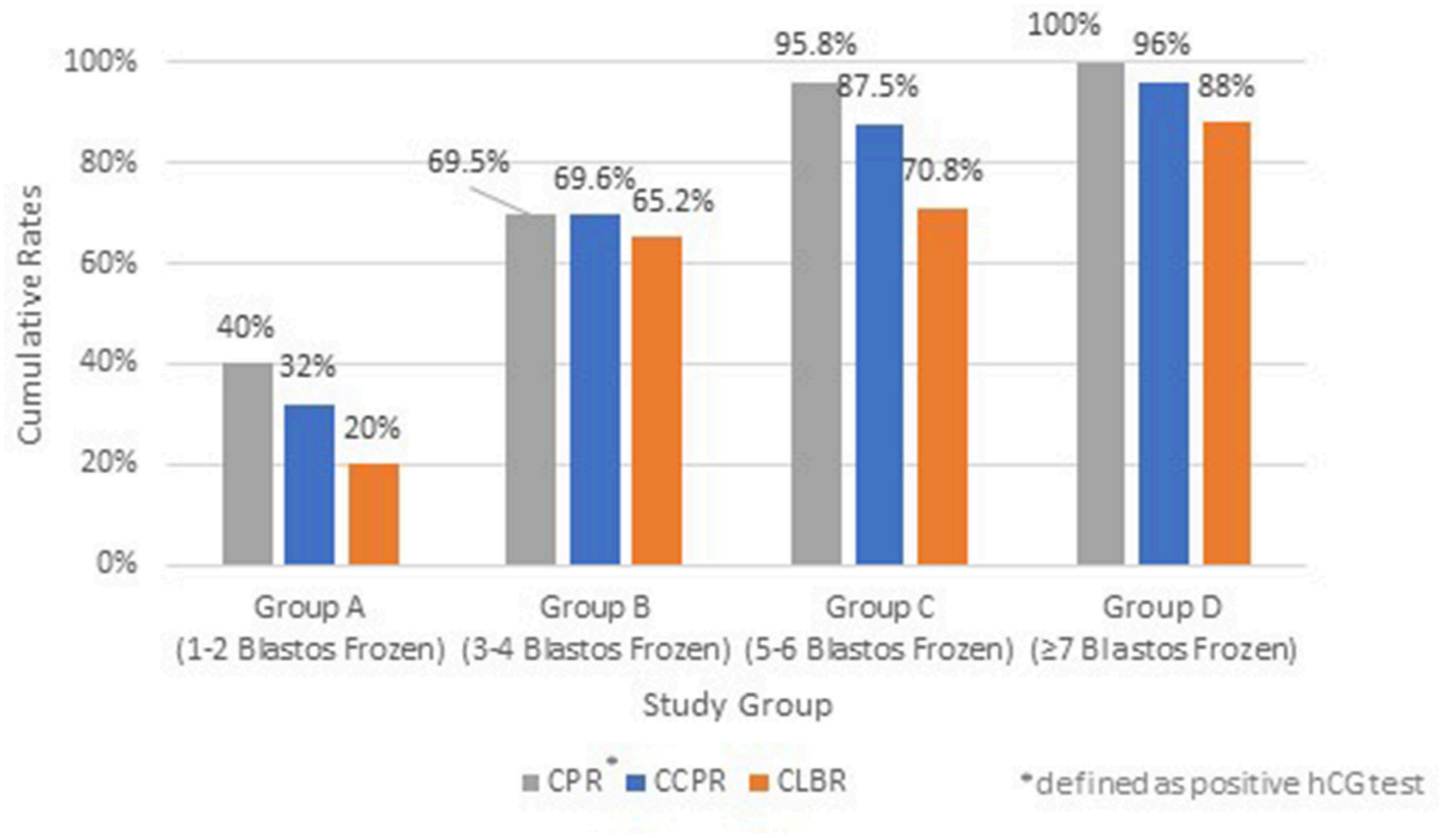
Cumulative Pregnancy Rate (CPR)^*^, Cumulative Clinical Pregnancy Rate (CCPR) and Cumulative Live Birth Rate (CLBR) of each study group; Group A (1–2 blastocysts frozen), Group B (3–4 blastocysts frozen), Group C (5–6 blastocysts frozen), and Group D (≥7 blastocysts frozen). ^*^Defined as positive hCG test.

The impact of the different number of available blastocysts for Freeze-all strategy after an ICSI cycle on CCPR and CLBR are summarized on Figure 3. Patients with 1–2 blastocysts vitrified achieved low CCPR of 32%, whereas the other groups showed 69.6% (Group B), 87.5% (Group C), and 96% (Group D), respectively, which is proven statistically significant (*p* < 0.05).

The CLBR for each group of patients are also depicted in Figure 3. The CLBR rose from 20%, when only 2 blastocysts were cryopreserved, up to 88% in group D with more than 7 blastocysts available (*p* < 0.05), taken into account that in this group some couples underwent second or third or even fourth embryotransfer in order to achieve such high CLBRs.

There were no incidents of triplets in the studied cases. The rate of twin deliveries was 33.3% (54/162) and the rate of the singleton deliveries was 66.7% (108/162) in the women of all groups (Table 3).

**Table 3:** The rate of singleton and twin deliveries in the live birth cases (N) in each group and in the overall live birth cases.

	**Group A (1–2 Blastos frozen) *N* = 10**	**Group B (3–4 Blastos frozen) *N* = 30**	**Group C (5–6 Blastos frozen) *N* = 34**	**Group D (≥7 Blastos frozen) *N* = 88**	**Overall *N* = 162**
Singleton delivery rate	80% (8/10)	66.7% (20/30)	59% (20/34)	68.2% (60/88)	66.7% (108/162)
Twin delivery rate	20% (2/10)	33.3% (10/30)	41% (14/34)	31.8% (28/88)	33.3% (54/162)

### Contribution of Each FET Cycle to the Cumulative Delivery Rate

**Figure 4:**
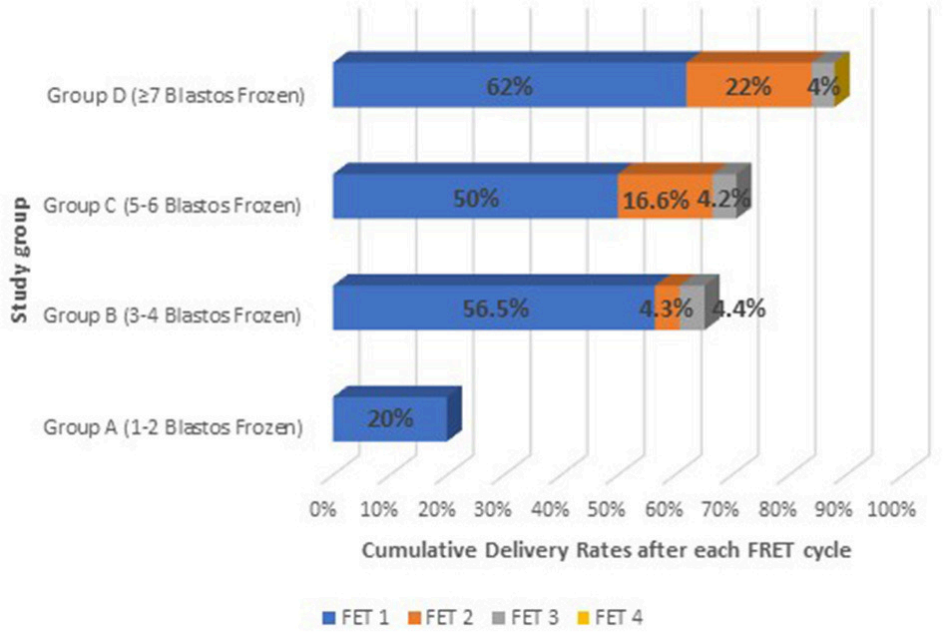
The cumulative effect on live birth after each FET cycle on all study groups: Group A (1–2 blastocysts frozen); Group B (3–4 blastocysts frozen); Group C (5–6 blastocysts frozen), and Group D (≥7 blastocysts frozen).

The cumulative increase in the delivery rates after each FET was calculated for each group of patients. The results of the cumulative effect of any additional FET cycle are presented on Table 4 and Figure 4.

**Table 4:** Live Birth Rate (LBR) after each FET cycle and Cumulative Live Birth Rate (CLBR) after each FET cycle of the studied groups according to the number of vitrified blastocysts.

	**LBR after FET 1**	**CLBR 1**	**LBR after FET 2**	**CLBR 2**	**LBR after FET 3**	**CLBR 3**	**LBR after FET 4**	**CLBR 4**	**Total CLBR**
Group A (1–2 Blastos Frozen) (*n* = 50)	20% (10/50)	**20%** (10/50)	0% (0/4)	**20%** (10/50)	N/A	N/A	N/A	N/A	**20%** (10/50)
Group B (3–4 Blastos Frozen) (*n* = 46)	56.5% (26/46)	**56.5%** (26/46)	14% (2/14)	**60.8%** (28/46)	100% (2/2)	**65.2%** (30/46)	N/A	N/A	**65.2%** (30/46)
Group C (5–6 Blastos Frozen) (*n* = 48)	50.0% (24/48)	**50.0%** (24/48)	30.7% (8/26)	**66.6%** (32/48)	50% (2/4)	**70.8%** (34/48)	N/A	N/A	**70.8%** (34/48)
Group D (≥7 Blastos Frozen) (*n* = 100)	62% (62/100)	**62%** (62/100)	64.7% (22/34)	**84%** (84/100)	33% (4/12)	**88%** (88/100)	0% (0/6)	88% (88/100)	**88%** (88/100)

*N/A, Not Applicable. The bold values represent the CLBR after each FET cycle*.

Group A had a delivery rate of 20% after the first frozen-thawed cycle, which was also the total cumulative rate, as the majority of the cases had embryos available for only one FET. In this group only 4 out of 50 patients performed single embryo transfer and therefore had a second FET. However, none of these FET treatments resulted in a delivery (these patients had decided to vitrify their embryos one by one).

Group B achieved 56.5% delivery rate after the first FET and the rate was increased by a 4.3% after the second FET cycle and additionally by 4.4% after the third one, reaching thus a CLBR of 65.2%. This increase in the delivery rate after the three sequential FET cycles is not considered significant (*p* > 0.05).

Group C showed an even higher increase in the Live Birth Rate after the second and the third FET. The delivery rate was significantly increased by 16.6% after the second FET (*p* < 0.05) and by only 4.2% after the third FET, reaching a total CLBR of 70.8%.

Group D was the only group which included patients with number of embryos available also for a fourth FET. Patients in that group had 62% delivery rate after the first FET, which was significantly increased by 22% (*p* < 0.05) after the second FET and by 4% after the third. The total CLBR for these patients was 88%. Despite that in this group there were 6 patients who had even a fourth frozen- thawed ET and although all six of them achieved a biochemical pregnancy in one out of the four transfers, finally none of them managed to deliver.

### Cumulative Live Birth Rates According to the Absolute Number of Vitrified Blastocysts

For reasons of further analysis, patients were also divided in 7 groups, according to the absolute number of vitrified blastocysts after the stimulation cycle; group I: 1-2 blastocysts frozen (*n* = 50), group II: 3 blastocysts frozen (*n* = 20), group III: 4 blastocysts frozen (*n* = 26), group IV: 5 blastocysts frozen (*n* = 26), group V: 6 blastocysts frozen (*n* = 22), group VI: 7 blastocysts frozen (*n* = 32) and group VII: more than 7 blastocysts frozen (*n* = 68).

**Figure 5:**
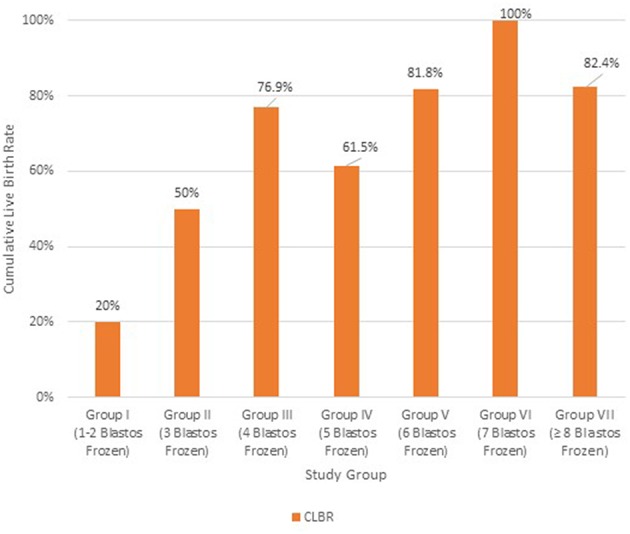
Cumulative Live Birth Rate (CLBR) in each group of patients according to the absolute number of vitrified blastocysts; Group I: 1–2 blastocysts frozen (*n* = 50); Group II: 3 blastocysts frozen (*n* = 20); Group III: 4 blastocysts frozen (*n* = 26); Group IV: 5 blastocysts frozen (*n* = 26); Group V: 6 blastocysts frozen (*n* = 22); Group VI: 7 blastocysts frozen (*n* = 32); Group VII: ≥8 blastocysts frozen (*n* = 68).

The Cumulative Live Birth Rates were further analyzed, according to the absolute number of vitrified blastocysts (Figure 5). Group I (1–2 blastocyst frozen) and Group II (3 blastocysts frozen) showed significantly different CLBRs of 20% (*n* = 50) and 50% (*n* = 20), respectively. Group III (4 blastocysts frozen) had higher rates of 76.9% (*n* = 26) and from this point and after the respective rates for the other groups with 5 (*n* = 26) and 6 (*n* = 22) blastocysts frozen did not differ statistically, as they were similar to that of Group III (61.5 and 81.8%, respectively). A significant increase in the CLBR was shown again when 7 blastocysts were available (*n* = 32). At this point the CLBR was 100% and the rate was significantly decreasing when more than 7 (*n* = 68) blastocysts were available (82.4%). In the latter case, the rates were similar with the CLBR of the group with 6 vitrified blastocysts (group V).

## Discussion

Embryo transfer in a fresh cycle, after the stimulation, has been for years the golden standard in IVF, despite the definite risk for OHSS, the definite negative effect on endometrial receptivity and despite the plausible negative impact on the achievement of a pregnancy. Looking from a cumulative delivery point of view, a fresh ET might sacrifice a euploid embryo in an unproper matured endometrium, whereas, a first time frozen embryo transfer may offer the best quality endometrium environment, increasing thus the cumulative probability of having a baby. The reason for not practicing all these years the segmentation of cycle in two parts (one fresh including stimulation, egg retrieval, fertilization and embryo culture and a second one including one or more thawed embryo transfers), was the modest survival rate after slow freezing, the even lower pregnancy rates after replacing thawed slow frozen embryos (37) and the inconclusive literature on the outcome of offsprings of frozen embryos (38, 39).

The emerging policy of Freeze-all is becoming more widely accepted as it is considered a safer treatment, especially for OHSS high risk patients (40) and under the wider acceptance and credibility that vitrification technique has gained (1, 18, 26, 37, 41–43), due to excellent post- thawing survival of the embryos and of the better synchrony of the endometrium cavity (10). Moreover, it has been suggested that Freeze-all strategy can lead to improved IVF results, when used individualized, according to each patient case (44).

The present study aimed to evaluate the efficacy of Freeze-all strategy in terms of Cumulative Live Birth Rates, according to the number of the blastocysts which were available for vitrification. For reasons of objectivity, the study did not include cases of poor responders, but only normal and high responders. Even patients of Group A, who had 1–2 blastocysts available for vitrification, were categorized as normal responders (5–24 oocytes retrieved). It should be noted that no embryo loss was observed in the studied cases, indicating the excellent efficacy of vitrification/ thawing process, at least in our hands.

It was expected that younger women especially with male infertility factor would form the group with high number of blastocysts. Nevertheless, such scenario was not the case in our sample. On the contrary, women of group A -having the lowest number of available blastocysts (1-2)- were the patients with the youngest mean age, which was surprisingly similar to the mean age of women of group D (more than 7 blastocysts available). The only statistically significant difference was observed between women of group A and group B., apparently by a matter of randomness.

In contrast to previous studies (8, 9, 23–25, 37, 45–48) which were based on the number of retrieved COCs to evaluate the success of an IVF treatment, the present research was designed upon the number of transferable blastocysts. Taking the decision -whether to transfer or segment- on the fifth day of embryo culture is more robust as the information provided about couple's implantation potential on that particular cycle is closer to reality. It is interesting that, in our analysis the mean number of the retrieved COCs differs significantly only in the case of group D (≥7 blastocysts) compared to all the other groups. Indeed, the number of the retrieved oocytes is similar between groups A, B and C. Although there is no significant difference in the oocyte number among these three groups, it is essential to focus on the clinical results that eventually rise form the different number of the formed blastocysts.

The results of the analysis have shown a significant increase in the Cumulative Live Birth Rates with the number of vitrified blastocysts. Indeed, patients in Group B achieved more than a triple rate of cumulative live birth compared to patients of Group A (20 vs. 65.2%, *p* < 0.05) and statistically similar results are obtained for Group C (70.8%). Unsurprisingly, the highest CLBR, as expected, was achieved by couples of Group D (88%), which had more than 7 blastocysts vitrified, but also were mostly high responders (Table 4).

The above results suggest that higher CLBRs can be expected when at least 3 blastocysts are formed. It appears that a total of three blastocysts is the threshold for achieving a CLBR of at least 50% (Figure 5). Obviously, live birth rates above 80% were noticed for patients with up to 6 vitrified blastocysts, implicating that at least 2 FET cycles are vital for higher clinical results. Indeed, for each extra 2 blastocysts available above two, the cumulative delivery rate was increasing. The extremely high cumulative live birth rate of 88% (Group D) when the couple segments the cycle and performs repetitive thawed embryo transfers, indicates first of all, the relative reproductive health of these couples, but also the potential of the Freeze-all strategy.

Patients of Group D (≥7 blastocysts frozen) achieved a live birth rate of 62% after the first FET and cumulatively after the third FET reached a level of 88% of live births. Although all women in this particular group had a positive hCG test, of those 12 who did not manage to deliver, four (33.3%) had a biochemical pregnancy and eight (66.6%) had a clinical pregnancy which ended as an abortion in <12 weeks. The above results indicate that high availability of transferable blastocysts might be related to better quality as well, and at the end those who did not give birth might be cases with other co-existing infertility factors related mainly to the mother.

Another interesting finding of our study is that although a significant increase exists in live birth rates between the first and the second FET, however, this effect is decreasing when a third or fourth FET cycle is required. This modest increase after a third embryo transfer (Figure 4) could possibly reflect the diminishing quality of the remaining embryos, as the morphologically best and thus most implantable blastocysts are usually preferred to be transferred first. Indeed, a fourth FET for 6 patients of Group D did not led to delivery, at any case.

On the contrary, the cumulative rates of Group A correspond to solely one FET cycle, limiting thus the possibility of a cumulative increase due to consequent frozen-thawed ETs. In this group, there was an exception of 4 patients who completed two single embryo transfers, as they had chosen to have their (two) embryos vitrified one by one. However, no clinical pregnancy was achieved in those cases. We have to underline that the fertilization rate of Group A was not statistically different, but the blastulation rate was significantly lower, comparing to the groups with higher number of blastocysts (Group C and Group D).

The conflicting results of previous studies do not support profoundly the prediction of the outcome of an IVF treatment based on the concept of the ovarian response of a woman (24, 49, 50). The present analysis proposes a new concept, which in contrary to previous research, is based on the blastulation rate, instead. The study of the number of the oocytes may reflect the ovarian response of a woman, but does not take into consideration other factors implicating in the embryo development. The results show that a more solid indicator on the potential efficacy of an ART cycle appears to be the blastulation rate, which reflects not only the impact of the pre-fertilization aspects (oocyte quality), but also of all the post- fertilization factors (sperm genetic quality, embryo genome activation, culture conditions, etc.). The combination of many parameters contributes to the formation of the blastocysts, which may have implantation potential. Thus, the ability of the zygotes to reach the blastocyst stage is the most promising indicator, regarding the clinical outcomes after a single oocyte retrieval.

Moreover, although Rubio et al. suggested that the percentage of the euploid embryos seems to decrease, when more oocytes are retrieved (51), others (52) support that a higher number of euploid embryos is observed when higher number of oocytes is retrieved. On the other hand, it has also been suggested that although the rate of euploid embryos remains stable, regardless the number of the available embryos (53, 54), every additional embryo significantly increases the chances of a woman to have at least one normal embryo (53). Consequently, the above literature agrees with our results, showing higher cumulative rates when more transferable blastocysts are available.

Adopting a Freeze-all strategy- besides the possible advantageous impact on the clinical results- is essential in preventing OHSS (40), especially in women with high oocyte yield, making the IVF treatment a safe procedure. Nevertheless, this strategy has not been proved beneficial for patients with less than two blastocysts available. A fresh embryo transfer in these cases might guarantee the same or even better results than the Freeze-all policy.

It is obvious, however, that the universal use of Freeze-all policy at the blastocyst stage, requires standardized and continuously controlled lab conditions, that may allow the best embryos to reach the ultimate *in vitro* developmental point. Moreover, an expertised lab personnel is essential to ensure successful vitrification and thawing procedures, that can guarantee embryo survival in a rate of more than 95%. Therefore, adopting strict lab quality control, any possible external factors that could affect the culture and the quality of the forming embryos, could be eliminated.

Another interesting finding was the high twin rate (>30%) in the last three groups (Table 3). This result indicates that since the cumulative delivery rate is really high, a viable option could be to implement elective single embryo transfer when more than 4 blastocysts are available, even if the time to delivery would prolongated with such policy. Since the cumulative delivery rate should remain equal, the twin rate would decrease dramatically to levels <1.5% and thus decreasing complications related with plausible preterm labors.

Currently, an indiscriminate submission of all IVF/ICSI patients to Freeze-all strategy and subsequent FET cycles is not supported by the clinical data. According to RCTs, Freeze-all is indeed a beneficial strategy for hyper- responders or patients who follow PGT-A at blastocyst stage, but may not necessary improve the clinical, obstetric or perinatal outcomes of the treatment. In contradiction, it may lead to increased treatment cost, lab workload, embryo manipulation and time to live birth (44). For this reason, the policy should be carefully and individually applied to each patient.

In conclusion, in order to answer the question which of the patients that follow Freeze-all policy, may benefit more in terms of cumulative live birth rates, our results suggest that the number of formed and vitrified blastocysts, can be an essential criterion to evaluate the eventual treatment outcome. The present study tried to analyze the eventual outcome of the treatment, irrespectively to the number of the produced oocytes, but based on a more solid evidence, as the number of the available blastocysts. However, more in-depth analysis is required in order to identify the subgroups of patients who really are meant for cryopreservation and those who are meant for fresh embryo transfers.

## Data Availability

All datasets generated for this study are included in the manuscript and/or the supplementary files.

## Author Contributions

All authors listed have made a substantial, direct and intellectual contribution to the work, and approved it for publication.

### Conflict of Interest Statement

The authors declare that the research was conducted in the absence of any commercial or financial relationships that could be construed as a potential conflict of interest.
